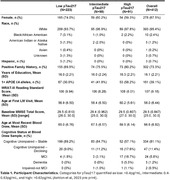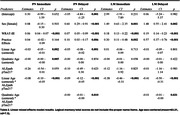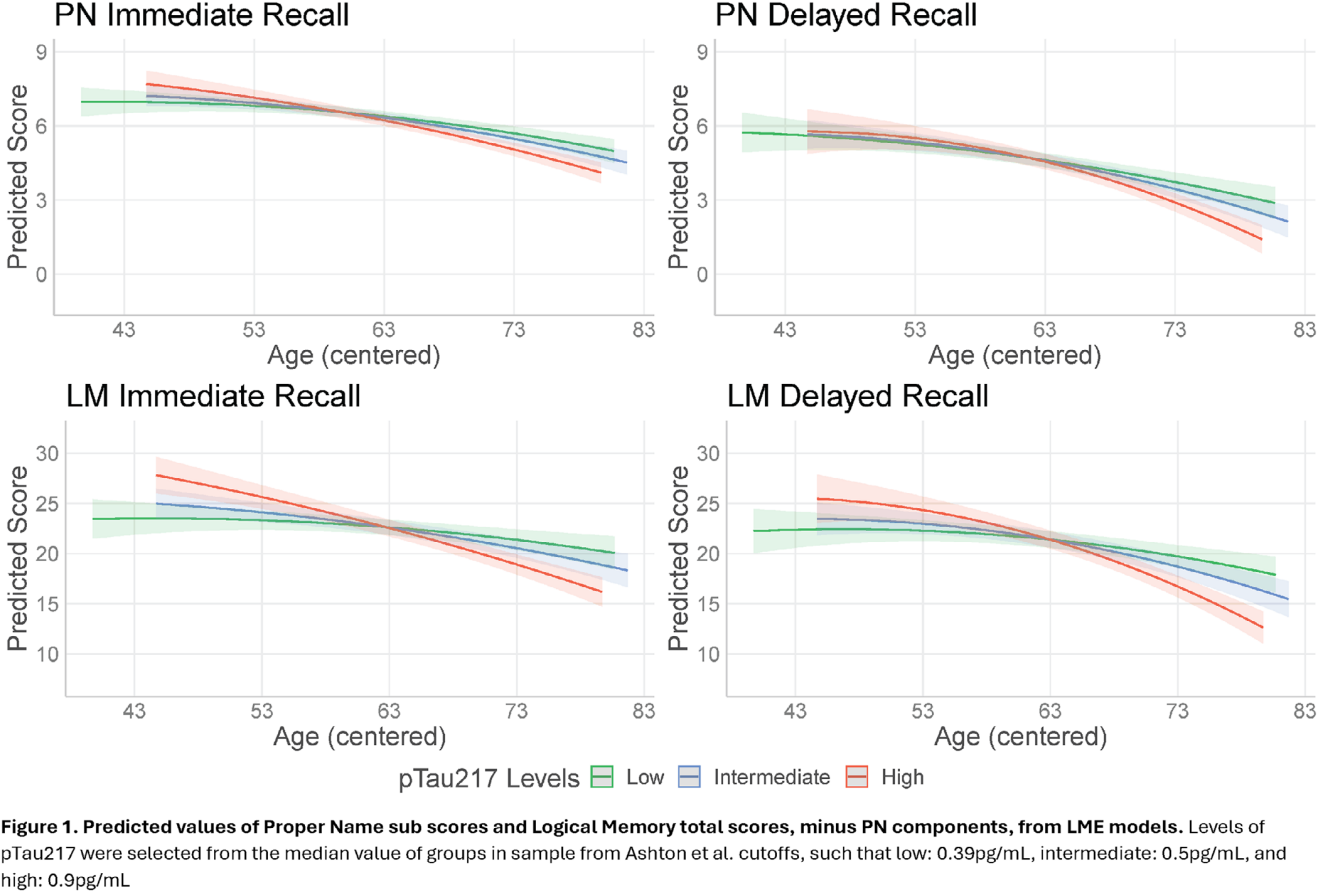# Longitudinal Change in Proper Names from Logical Memory Tasks are Sensitive to Plasma pTau217 Levels

**DOI:** 10.1002/alz.087520

**Published:** 2025-01-09

**Authors:** Kristin E Basche, Rebecca E. Langhough, Erin M. Jonaitis, Lianlian Du, Davide Bruno, Rachael E Wilson, Bruce P Hermann, Sterling C. Johnson, Kimberly D Mueller

**Affiliations:** ^1^ Department of Medicine, University of Wisconsin‐Madison School of Medicine and Public Health, Madison, WI USA; ^2^ Wisconsin Alzheimer's Disease Research Center, University of Wisconsin‐Madison, School of Medicine and Public Health, Madison, WI USA; ^3^ Alzheimer’s Disease Research Center, University of Wisconsin‐Madison School of Medicine and Public Health, Madison, WI USA; ^4^ Wisconsin Alzheimer’s Institute, University of Wisconsin‐Madison School of Medicine and Public Health, Madison, WI USA; ^5^ Department of Biostatistics and Medical Informatics, School of Medicine and Public Health, University of Wisconsin‐Madison, Madison, WI USA; ^6^ Wisconsin Alzheimer's Disease Research Center, Madison, WI USA; ^7^ The Wisconsin Alzheimer's Institute, Madison, WI USA; ^8^ Liverpool John Moores University, Liverpool United Kingdom; ^9^ Wisconsin Alzheimer's Disease Research Center, University of Wisconsin School of Medicine and Public Health, Madison, WI USA; ^10^ Department of Medicine, Division of Geriatrics and Gerontology, School of Medicine and Public Health, University of Wisconsin–Madison, Madison, WI USA; ^11^ Wisconsin Alzheimer's Disease Research Center, School of Medicine and Public Health, University of Wisconsin‐Madison, Madison, WI USA; ^12^ Wisconsin Alzheimer's Institute, Madison, WI USA; ^13^ University of Wisconsin‐Madison, School of Medicine and Public Health, Madison, WI USA; ^14^ VA Geriatric Research, Education and Clinical Center (GRECC), William S. Middleton Memorial Veterans Hospital, Madison, WI USA; ^15^ Waisman Center, University of Wisconsin‐Madison, Madison, WI USA; ^16^ Alzheimer's Disease Research Center, University of Wisconsin‐Madison, Madison, WI USA; ^17^ Geriatric Research Education and Clinical Center, William S. Middleton Memorial Veterans Hospital, Madison, WI USA; ^18^ Wisconsin Alzheimer’s Institute, University of Wisconsin School of Medicine and Public Health, Madison, WI USA; ^19^ School of Medicine and Public Health, University of Wisconsin‐Madison, Madison, WI USA; ^20^ Department of Communication Sciences and Disorders, University of Wisconsin‐Madison, Madison, WI USA

## Abstract

**Background:**

Previous studies have found connections between recall of proper names (PN) and amyloid positivity in cognitively unimpaired (CU) adults at risk for Alzheimer’s Disease (AD; Mueller et al., 2020). Given the promising prospect of employing plasma‐based biomarkers to determine amyloid burden, we looked at associations between longitudinal change in PN and total score recall from Logical Memory (LM) story recall test and plasma pTau217.

**Method:**

Participants from the Wisconsin Registry for Alzheimer’s Prevention (WRAP) study, who were CU at baseline LM visit; had item‐level, longitudinal LM and plasma pTau217 data from a recent blood draw (n=412; EDTA plasma samples analyzed using the ALZpath pTau217 Simoa assay on a Quanterix HD‐X). Linear‐mixed effects models were utilized to examine whether the last available plasma pTau217 measure moderated retrospective age (centered at mean=63) trajectories associated with LM Total Score and PN subscores; pTau217 interactions with quadratic age were removed if they were non‐significant. Models included a person‐level random intercept term, sex, practice effects, and WRAT‐III reading standard scores as covariates.

**Results:**

Sample characteristics overall and by last observed plasma pTau217 category (Ashton et al, 2023 pre‐print) are shown in Table 1. The sample was mostly female (n=278, 67.5%), and white (n=393, 95.4%), with an average age of 58 (sd=6.44) at baseline visit. The interaction term of pTau217 was significant for all of the models for linear age (Table 2, β ranging from ‐0.50 through ‐0.08, p<0.001); the interaction of pTau217 was also significant for quadratic age in the PN delayed subscore (β=‐0.00, p=0.010) and the LM delayed total score (β=‐0.01, p=0.031).

**Conclusion:**

Change in LM total and PN subscores are sensitive to amyloid burden via plasma pTau217. Participants with high levels of pTau217 performed more poorly over time than those with intermediate or low levels (Figure 1). Despite being limited to only nine items on LM tasks, PN subscores continue to demonstrate their strength as a potential indicator of amyloid accumulation as measured by a quick and lower‐burden blood test.